# Differential Impact of Methamphetamine Dependence and Social Media Overuse on Cognitive Control: Based on the Dual Mechanisms of Control Theory

**DOI:** 10.3390/bs15081086

**Published:** 2025-08-12

**Authors:** Meng Zhang, Xikun Zhang, Tiange Xu, Jifan Zhou, Mowei Shen

**Affiliations:** Department of Psychology and Behavioral Sciences, Zhejiang University, Hangzhou 310058, China; zhang.meng@zju.edu.cn (M.Z.); xikun_zhang@zju.edu.cn (X.Z.); xutiange@zju.edu.cn (T.X.)

**Keywords:** cognitive control, methamphetamine dependence, social media overuse, working memory capacity, reactive control, proactive control

## Abstract

Cognitive control impairments contribute to the onset and maintenance of both substance and behavioral addictions. Guided by the Dual Mechanisms of Control framework, this study examined cognitive control deficits in methamphetamine-dependent individuals and those who overuse social media, each compared to a matched control group. Across two experiments, participants completed an operational working memory span task (Experiment 1) to assess their cognitive control resources, and a modified AX-Continuous Performance Test (AX-CPT, Experiment 2) to evaluate their inhibition-based proactive and reactive control. The results indicated that while both methamphetamine-dependent individuals and social media overusers demonstrated cognitive control impairments, the severity, affected components, and overall patterns differed. Methamphetamine-dependent individuals were characterized by more pronounced, pervasive deficits and a maladaptive reliance on compromised reactive control. In contrast, social media overuse was associated with milder impairments, maintaining relatively intact proactive versus reactive control patterns. These findings underscore the distinct cognitive control profiles underlying substance versus behavioral addictions and highlight the necessity of developing tailored intervention strategies to address the specific cognitive vulnerabilities of each population.

## 1. Introduction

Cognitive control serves as a fundamental mechanism for effective daily functioning, coordinating higher-order cognitive processes that oversee perception, attention, and behavioral responses in pursuit of overarching goals (Banich, 2009; Miyake & Friedman, 2012). The ability to regulate thoughts and actions according to internal goals is essential for adaptive behavior across diverse life contexts, from academic and professional performance to social relationships and personal well-being. However, this critical capacity becomes significantly compromised in individuals with addictive disorders. Extensive research has demonstrated that both substance and behavioral addictions are characterized by impairments in cognitive control, though these deficits manifest to varying degrees and potentially through different mechanisms (Dalley et al., 2011; Dean et al., 2013; Feil et al., 2010; Moccia et al., 2017). While substantial evidence points to commonalities between substance addiction and behavioral addiction (Alavi et al., 2012; Grant et al., 2010; Robbins & Clark, 2015), emerging theoretical perspectives suggest that distinct neurobiological and psychological mechanisms may underlie substance-based addictions compared to those driven primarily by behavioral patterns (Koob & Volkow, 2010; Potenza, 2006; Robbins & Everitt, 1996; Volkow et al., 2003). Despite this theoretical distinction, empirical research directly comparing cognitive control profiles across different addiction types remains limited, creating a critical gap in our understanding of the mechanisms that differentiate various addictive processes.

According to the unity and diversity framework, cognitive control demonstrates both shared commonalities and distinct specificities, with three core components consistently identified across empirical studies: updating (working memory), shifting (cognitive flexibility), and inhibiting (inhibitory control) (Friedman & Miyake, 2017). These executive functions are unified by a common underlying mechanism—the ability to actively maintain and manipulate goal representations to guide the top-down control of cognition and behavior (Gavazzi et al., 2023b; Jentsch & Pennington, 2014; Volkow et al., 2011). However, each component also contributes unique variance to cognitive performance, reflecting specialized processes that support different aspects of controlled behavior. Working memory serves as the foundational resource system, providing the capacity to maintain task-relevant information in an active state while managing interference from irrelevant stimuli (Wiemers & Redick, 2018). Inhibitory control represents the core regulatory mechanism, enabling the suppression of prepotent or inappropriate responses in favor of goal-directed actions (Friedman & Miyake, 2017). This intricate interplay between working memory and inhibitory control forms the cornerstone of adaptive cognitive functioning and goal achievement.

The integrity of these cognitive control processes is essential for maintaining adaptive behavior across diverse contexts. When working memory and inhibitory control systems become compromised, individuals may experience significant difficulties in regulating their thoughts and actions according to long-term goals (Kane & Engle, 2002; Miyake et al., 2000). As the foundational resource of cognitive control, working memory facilitates the coordinated operation of executive functions by providing the cognitive resources necessary for inhibitory control and cognitive flexibility to operate effectively (Engle & Kane, 2004; Wiemers & Redick, 2018). Extensive empirical evidence has demonstrated strong correlations between working memory capacity and various measures of cognitive control, including inhibitory control efficiency, task-switching performance, and conflict resolution abilities (Friedman & Miyake, 2004; Redick, 2014; Unsworth & Spillers, 2010). This robust relationship reflects the fundamental role of working memory as the cognitive architecture that enables the maintenance of goal representations while managing competing cognitive demands. People with substance and behavioral addictions have been found to manifest impaired working memory capacity (Bechara & Martin, 2004), which in turn compromises their cognitive control capabilities and contributes to difficulties in regulating addictive behaviors (Goldstein & Volkow, 2011; Wiemers & Redick, 2018). Notably, these working memory deficits appear to have particularly pronounced effects on inhibitory control processes, as the limited capacity to maintain goal-relevant information directly undermines the ability to suppress inappropriate responses and resist immediate temptations.

Building on this understanding of working memory’s role in supporting cognitive control, inhibitory control has emerged as particularly critical for adaptive behavior and especially vulnerable to the effects of working memory limitations. Inhibitory control enables individuals to override prepotent responses and resist distractions when pursuing goal-directed actions (Diamond, 2013; Friedman & Miyake, 2017). Inhibitory control operates through the suppression of inappropriate thoughts, emotions, or behaviors that conflict with current goals, thereby allowing for flexible and contextually appropriate responding (Miyake et al., 2000; Nigg, 2000). This executive function is especially vulnerable in clinical populations, where deficits in inhibitory control have been consistently linked to difficulties in self-regulation and maladaptive behaviors (Jentsch & Pennington, 2014; Volkow et al., 2011). To better understand the mechanisms underlying inhibitory control, Braver’s (2012) Dual Mechanisms of Control (DMC) theory provides a comprehensive framework that distinguishes between two distinct modes of cognitive control: proactive control and reactive control. Proactive control is characterized by the sustained maintenance of goal-related information to guide behavior in a predictive, anticipatory manner, involving sustained activation in rostral and lateral regions of the prefrontal cortex (PFC) (Gavazzi et al., 2019, 2023a). Reactive control, in contrast, is activated by the detection of conflicts or changes in the environment and relies on transient, corrective adjustments, associated with transient activation in more dorsal and caudal PFC regions (Friedman & Robbins, 2022; Gavazzi et al., 2019, 2023a). Critically, these two forms of cognitive control with their embedded inhibitory processes are central to understanding addiction-related deficits. Proactive control engages proactive inhibition—the anticipatory suppression of potential inappropriate responses before they become activated, allowing individuals to prevent interference from arising (Schröder et al., 2024). Reactive control engages reactive inhibition—the halting of already-initiated inappropriate responses when conflicts are detected, requiring rapid suppression of ongoing response tendencies (Bravi et al., 2022; Gavazzi et al., 2021). 

The DMC framework has particular relevance for understanding different types of addiction, as the distinct etiological mechanisms underlying substance versus behavioral addictions may differentially affect proactive and reactive control processes. Individuals with sufficient cognitive resources can flexibly engage both control modes depending on contextual demands (Braver, 2012; Chiew & Braver, 2017). However, individuals with limited cognitive resources may rely more heavily on reactive control to conserve cognitive resources, though this often results in difficulties maintaining systematic, goal-directed behavior (Duncan et al., 1996; Kane & Engle, 2002). Substance addiction typically involves direct pharmacological effects on neural pathways, particularly affecting dopaminergic systems and prefrontal regions critical for cognitive control (Koob & Volkow, 2010; Volkow et al., 2003). These neurobiological alterations may primarily compromise the sustained neural activity required for proactive control, forcing greater reliance on reactive control mechanisms. In contrast, behavioral addictions may develop through different pathways, potentially involving altered reward learning and habit formation without the direct pharmacological disruption of cognitive control networks (Everitt & Robbins, 2016; Volkow & Morales, 2015). Such mechanistic differences suggest that substance and behavioral addictions may exhibit distinct patterns of proactive and reactive control impairments, yet direct empirical comparisons remain scarce in the literature.

Among various substance addictions, methamphetamine dependence represents a particularly severe form that has reached epidemic proportions globally. In China, methamphetamine has become the most commonly abused synthetic substance according to the “China Drug Situation Report 2023,” leading to significant social, familial, and personal health consequences (Dean et al., 2013; Rubenis et al., 2018). Methamphetamine’s potent effects on dopaminergic and noradrenergic systems make it an ideal model for studying substance-induced cognitive control deficits. Concurrently, the digital age has brought attention to social media overuse as a prominent behavioral addiction (Andreassen, 2015). With the widespread adoption of social media platforms, excessive usage has been associated with compulsive behaviors and withdrawal symptoms that parallel those observed in substance addictions (Andreassen, 2015; Kuss & Griffiths, 2017; Montag et al., 2018). Neuroimaging research suggests that both addiction types involve overlapping neurobiological alterations affecting the cognitive functions crucial for daily life and decision-making (Grant et al., 2010). However, the specific patterns of cognitive control impairments and their underlying mechanisms may differ significantly between these addiction types, reflecting their distinct etiological pathways.

The present study aims to provide a systematic comparison of cognitive control profiles between methamphetamine dependence and social media overuse through two complementary experiments. Experiment 1 employed an operational working memory span task (Conway et al., 2005) to assess working memory capacity differences across groups. Experiment 2 utilized an adapted AX-Continuous Performance Test (AX-CPT, Cooper et al., 2017) to examine proactive versus reactive control patterns according to the DMC framework.

We propose that while both methamphetamine-dependent individuals and social media overusers may exhibit impaired working memory and diminished proactive/reactive control, the severity and patterns of these deficits are expected to differ. These differences likely reflect the distinct psychological mechanisms underlying substance and behavioral addictions.

## 2. Experiment 1 Capacities of Working Memory in Methamphetamine-Dependent Individuals and Social Media Overusers

### 2.1. Method

#### 2.1.1. Participants

To ensure adequate statistical power for detecting group differences, we conducted an a priori power analysis using G*Power (version 3.1). Based on an effect size of *f* = 0.40 (Ersche et al., 2006) and using α = 0.05 with a desired power of 0.90. we conducted a sample size calculation for an ANCOVA with two groups and one covariate (age for MDG vs. MCG and gender for SMG vs. SCG). The analysis indicated that a total sample size of 68 participants (34 per group) would be sufficient to detect a significant group effect under these conditions.

The experiment comprises four groups: methamphetamine-dependent (MDG, n = 43, all males) and its control group (MCG, n = 43, all males), and social media overuse (SMG, n = 40, 21 females) and corresponding control group (SCG, n = 73, 30 females). These sample sizes exceeded the a priori requirement, ensuring that the study was adequately powered for the reported analyses. Detailed demographic and clinical information for these participants is presented in Table 1.

MDG versus MCG: Male individuals with a history of methamphetamine addiction, currently in the rehabilitation phase after physiological detoxification, were recruited from a compulsory drug rehabilitation center in Zhejiang Province, China. As the center only admits men, both the methamphetamine-dependent group and its control group (recruited with payment) consisted exclusively of male participants, ensuring gender consistency in the study.

SMG versus SCG: We administered a screening questionnaire to college students to recruit social media overuse participants and corresponding control participants. The group characterized by excessive social media use was identified using an adapted version of the Internet Addiction Test (IAT, Young, 2004), with scores above 66 points, and the adapted Bergen Facebook Addiction Scale (BFAS, Andreassen et al., 2012) with scores of at least 56 points. Details of the selection process and criteria are provided in the Supplementary Materials. In addition to meeting the thresholds set by these scales, participants must also fulfill the criteria of engaging with social media at least 5 to 7 days per week, with daily usage exceeding 4 hours.

Participants in the four groups are all right-handed and over 18 years old; they have not experienced a major trauma recently and have no history of mental illness. SMG and the two control groups should have no history of methamphetamine or other illicit drug use, nor should they have used any medications that affect the central nervous system. 

This research strictly followed the principles outlined in the Declaration of Helsinki and received ethical approval from Zhejiang University.

#### 2.1.2. Apparatus

The experiment task was programmed by MATLAB (R2017b) software in conjunction with the Psychophysics Toolbox extension, as detailed by Brainard (1997) and Pelli (1997). The stimuli were displayed on a 24-inch LCD monitor with a resolution of 1920 × 1080 pixels. Participants were seated at a calibrated distance of approximately 60 centimeters from the monitor corresponding to a full-screen visual angle of roughly 47.78° horizontally by 27.98° vertically. Experimental manipulations and responses were facilitated through the use of a standard keyboard.

#### 2.1.3. The Operant Working Memory Span Task

**Figure 1:**
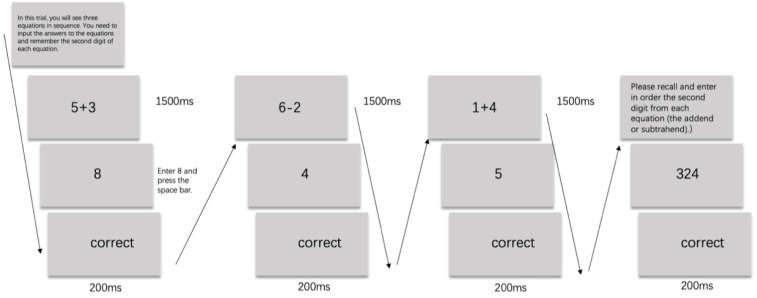
The Operational Working Memory Span Task used in Experiment 1.

We examined the working memory capacities of our participants using the Operant Working Memory Span Task (Conway et al., 2005) to discern potential differences in cognitive control abilities. In this task, participants consecutively performed n rounds of single-digit addition and subtraction operations, concurrently maintaining continuous memory of the second digit in each arithmetic sequence. After completing the calculations, they were required to recall the target digits of the n operations in sequence before advancing to the next trial (Conway et al., 2005). The participant’s working memory span score is recorded as n − 1, where n is the level at which the participant made their first error, meaning the score is based on the last level completed correctly. A higher score indicates greater working memory capacity. The experimental procedure is illustrated in Figure 1.

#### 2.1.4. Results

**Figure 2:**
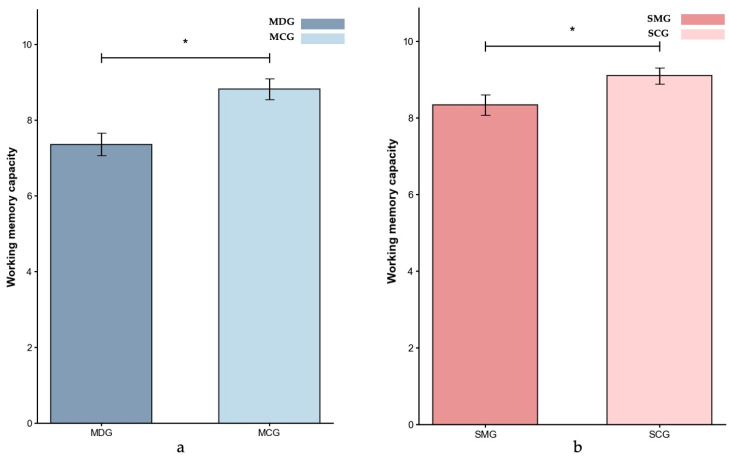
Working memory capacity (*M ± SE*) for the MDG and MCG groups (**a**) and the SMG and SCG groups (**b**) in Experiment 1. Error bars represent ±1 standard error of the mean, *p* < 0.05: *.

Eventually, valid data were obtained from 199 participants in the experiment 1. The operational working memory span task results for the MDG/MCG and SMG/SCG groups are presented in Figure 2.

Given the significant age difference between the MDG (*M* = 35.30 years, *SD* = 4.93 years) and MCG (*M* = 24.12 years, *SD* = 2.36 years) groups (*p* < 0.001), and considering that age is a critical factor influencing cognitive control (Braver & Barch, 2002; Verhaeghen & Cerella, 2002), we conducted the ANCOVA with age as a covariate to assess group differences in working memory span. The result indicated that the MDG group (*M* = 7.30) had significantly lower working memory capacity than the MCG group (*M* = 8.91), *p* = 0.048, partial *η*^2^ = 0.046.

For the SMG and SCG comparison, with groups being age-matched (*p* > 0.05), gender was included as a covariate in the ANCOVA. The result showed that participants in the SMG group (*M* = 8.40) had a significantly lower working memory capacity than those in the SCG group (*M* = 9.08), *p* = 0.041, partial *η*^2^ = 0.037.

Comparing the effect sizes across groups revealed a slightly greater impairment in the MDG group (partial *η*^2^ = 0.046) than in the SMG group (partial *η*^2^ = 0.037), indicating that while both groups exhibited a reduced working memory capacity relative to their respective control groups, the deficit was more pronounced among individuals with methamphetamine dependence.

## 3. Experiment 2 Proactive and Reactive Control in Methamphetamine-Dependent Individuals and Social Media Overusers

### 3.1. Method

#### 3.1.1. Participants

Experiment 2 comprises four groups too: methamphetamine dependence (MDG, n = 43) and its control group (MCG, n = 38), and social media overuse (SMG, n = 42, 31 females) and its corresponding control group (SCG, n = 55, 17 females). The participants were all newly recruited in Experiment 2, with identical selection criteria to those in Experiment 1. Consistent with the a priori power analysis conducted using G*Power, these sample sizes met the calculated requirement to ensure there was adequate power for detecting group differences under the study conditions. Detailed demographic and clinical information for these participants is presented in Table 2.

#### 3.1.2. Apparatus

The experimental environment and equipment were identical to those used in Experiment 1.

#### 3.1.3. AX-Continuous Performance Test (AX-CPT)

**Figure 3:**
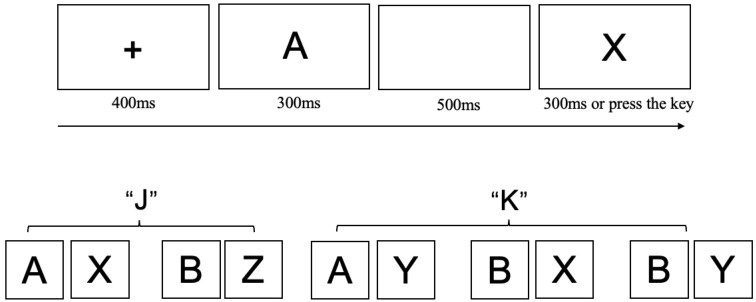
The Adapted AX-CPT Task used in Experiment 2. Each trial began with a central fixation cross (“+”) presented for 400 ms, followed by a cue letter at the center of the screen. After a 500 ms blank screen, a probe letter was shown for 300 ms or until the participant responded. Participants were instructed to press the “J” key if the cue was “A” followed by probe “X”, or if the cue was “B” followed by probe “Z” (the “AZ” combination did not exist), press the “K” key if the cue was “A” followed by probe “Y”, or if the cue was “B” followed by probe “X” or “Y”.

We utilized a modified AX-CPT task (Cooper et al., 2017) to assess the levels and patterns of proactive and reactive control while minimizing the likelihood of participants adopting strategic shortcuts. Details of the modifications are provided in the Supplementary Materials, and the experimental procedure is illustrated in Figure 3. In this adapted version, participants responded to target sequences (AX and BZ) and non-target sequences (AY, BX, BY). 

The task predominantly consists of AX trials, comprising 70% of the total, which conditions participants to anticipate a target response when the cue “A” is followed by the probe “X”. This setup is intended to measure both proactive control, where participants prepare a response based on the cue, and reactive control, where they rely on the late-presenting probe to adjust their behavior. Regarding performance indices, the AX trials serve as an Index of habituated response, since participants are conditioned to expect a target response following the “A” cue. The AY trials represent an Index of reactive control, as participants need to inhibit their automatic target response when the probe does not match the expected “X”. The BX trials are used as an Index of proactive control, requiring participants to override the response tendency when the probe “X” is presented. The BZ trials, in which the second letter is always “Z” require participants to make a target judgment regardless of the first letter. This design ensures that even when the first letter is “B” (rather than “A”), participants cannot preemptively classify the sequence as a non-target and are instead required to wait for the second letter before making a response. The inclusion of BZ trials addresses the potential confound of participants adopting a strategy where they automatically prepare for a ‘non-target’ judgment upon encountering the first letter “B” bypassing further processing of the second letter. Such a strategy would undermine the role of the probe “X” as a target stimulus, thereby compromising the validity of the experimental design. Finally, BY trials function as an Index of baseline response levels, as they do not involve conflict between cue and probe, providing a measure of the participants’ baseline cognitive performance without strong expectancy or inhibition requirements (The specific trial distribution and definitions of the indices refer to Table 3).

#### 3.1.4. Results

Prior to analysis, we used MATLAB (R2017b) to apply a within-subject, within-condition trimming procedure, removing outlier trials with reaction times exceeding ±3 standard deviations from each participant’s mean for each condition (Rousseeuw & Croux, 1993). Participants with an excessive proportion of outlier trials, resulting in insufficient valid trials for reliable estimation of condition-level performance (fewer than 50% of trials retained per condition, Ratcliff, 1993), were excluded from further analysis. Following these procedures, valid data were obtained from 178 participants out of the initial 181.

The primary focus of the analysis in Experiment 2 was on two key metrics: the Inverse Efficiency Scores (IES; Liesefeld & Janczyk, 2019) in different trial conditions (BY, BX, AY) and the Proactive Behavioral Index (PBI; Cooper et al., 2017).

The IES was calculated as the ratio of accuracy to reaction time, where a higher IES value indicates better performance (i.e., faster responses with higher accuracy). Meanwhile, the PBI reflects participants’ propensities for proactive versus reactive control. To assess the relative inclination towards proactive versus reactive control, the *PBI* was calculated, utilizing the following formula: $PBI=\frac{{IES}_{BX}-{IES}_{AY}}{{IES}_{AY}+{IES}_{BX}}$, where a positive index indicates superior performance in BX relative to AY trials, suggesting a greater reliance on proactive control, and a negative index indicates the opposite, implying greater dependence on reactive control. ANCOVAs were conducted to compare *IES* and *PBI* scores between the experimental and control groups (MDG/MCG, SMG/SCG). Age was included as a covariate in the analysis for the MDG/MCG groups, while gender was included as a covariate in the analysis for the SMG/SCG groups. 

**Figure 4:**
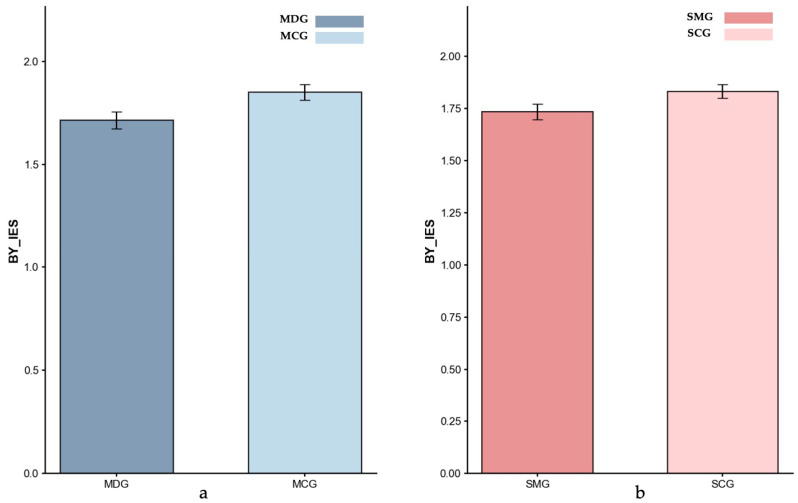
Inverse Efficiency Scores (IES) for BY-baseline: (**a**) MDG and MCG, and (**b**) SMG and SCG. Error bars represent ±1 standard error of the mean.

**Figure 5:**
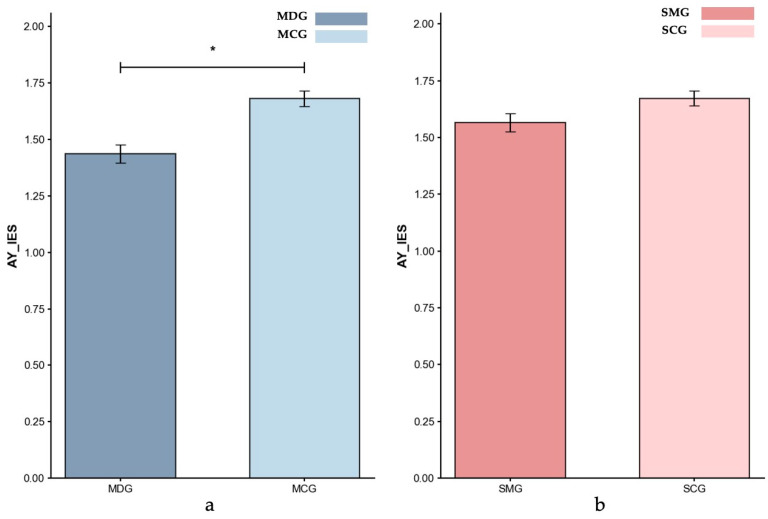
Inverse Efficiency Scores (IES) for AY-Reactive control: (**a**) MDG and MCG, and (**b**) SMG and SCG. Error bars represent ±1 standard error of the mean, *p* < 0.05: *.

**Figure 6:**
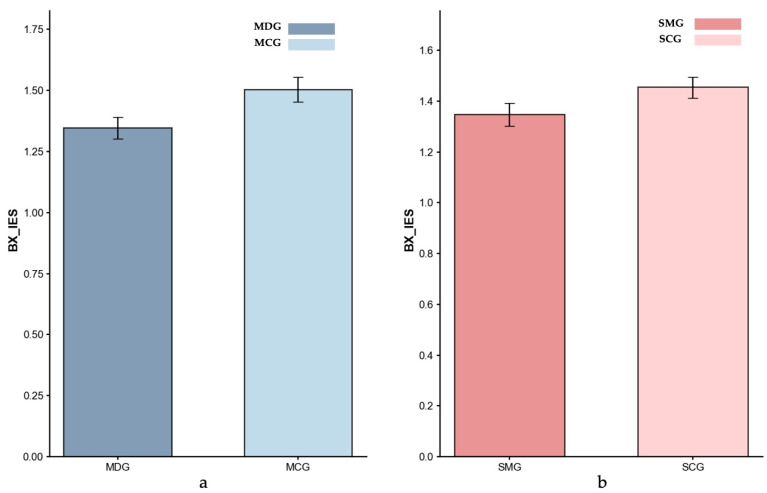
Inverse Efficiency Scores (IES) for BX-Proactive control: (**a**) MDG and MCG, and (**b**) SMG and SCG. Error bars represent ±1 standard error of the mean.

Inverse efficiency scores (IES). Figure 4, Figure 5 and Figure 6 present the IES index data for all four groups across different trial types.

*Baseline (BY Trials)*. Based on the ANCOVA result controlling for age, no significant difference was found between the MDG (*M* = 1.71) and MCG (*M* = 1.85) groups, *p* = 0.191, partial *η^2^* = 0.022. These findings indicated that the MDG and MCG groups exhibited a comparable baseline performance. Similarly, an ANCOVA with gender as a covariate indicated no significant difference between the SMG (*M* = 1.74) and SCG *(M* = 1.83) groups; however, the result approached significance (*p* = 0.081, partial *η*^2^ = 0.032), suggesting a trend toward reduced baseline performance in the SMG group.

*Reactive Control (AY Trials)*. Controlling for age, the ANCOVA result showed that the MDG exhibited a significantly lower reactive IES (*M* = 1.44) compared to the MCG (*M* = 1.68; *p* = 0.017, partial *η*^2^ = 0.071), indicating poorer reactive control performance in the MDG. Additionally, an ANCOVA controlling for gender showed that the SMG (*M* = 1.56) exhibited a trend toward lower reactive control performance compared to the SCG (*M* = 1.67), with the difference approaching significance (*p* = 0.056, partial *η*^2^ = 0.038). Comparing the effect sizes across groups revealed a larger impairment in reactive control in the MDG group than in the SMG group, highlighting the more pronounced disruption associated with methamphetamine dependence.

*Proactive Control (BX Trials).* Controlling for age, the ANCOVA result showed no significant difference in proactive IES between the MDG (*M* = 1.35) and the MCG (*M* = 1.50; *p* = 0.201, partial *η*^2^ = 0.021), indicating comparable performance between the two groups under the proactive control condition. Similarly, an ANCOVA with gender as a covariate indicated that the SMG’s IES index (*M* = 1.35) was not significantly different from the SCG’s (*M* = 1.45; *p* = 0.120, partial *η*^2^ = 0.026). 

**Figure 7:**
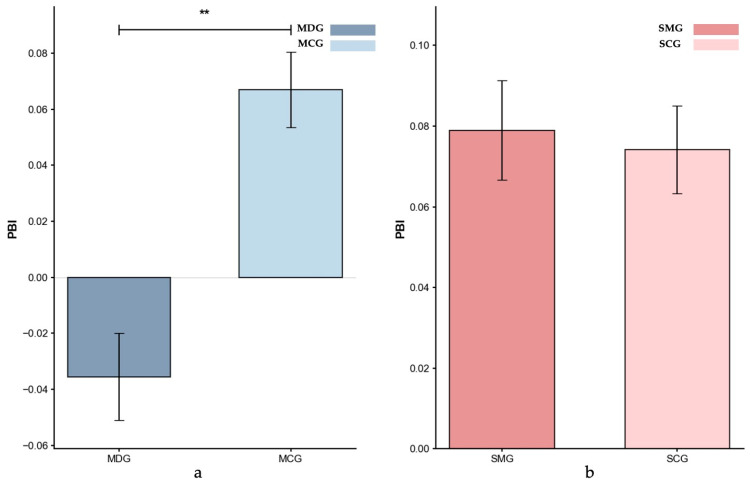
Proactive Behavioral Index (PBI): (**a**) MDG and MCG, and (**b**) SMG and SCG. Error bars represent ±1 standard error of the mean, *p* < 0.01: **.

*Proactive Behavioral Index (PBI)*. Figure 7 displays the mean PBI scores for the four groups. Controlling for age, the ANCOVA result showed that the MDG had significantly lower PBI scores compared to the MCG group (MDG: *M* = −0.035; MCG: *M* = 0.067; *p* = 0.009, partial *η*^2^ = 0.083), indicating a much greater reliance on reactive control in the MDG. In contrast, an ANCOVA, controlling for gender, showed no significant difference between the SMG (*M* = 0.079) and SCG (*M* = 0.074) groups (*p* = 0.446, partial *η*^2^ = 0.001), suggesting similar reliance on proactive versus reactive control mechanisms for SMG participants. 

## 4. Discussion

Drawing on Friedman and Miyake’s (2017) unity/diversity model of executive functions and Braver’s (2012) Dual Mechanisms of Control framework, this study examined the cognitive control mechanisms underlying substance addiction (methamphetamine-dependent) and behavioral addiction (social media overuse). Our findings provide empirical support for the theoretical distinction between substance and behavioral addictions, demonstrating that while both exhibit impairments in the foundational cognitive control resources—working memory—they differ significantly in the severity, pattern, and specific control mechanisms affected. These differential patterns align with the DMC framework’s prediction that distinct etiological mechanisms should manifest as different profiles of proactive and reactive control impairments.

Both groups demonstrated significant deficits in working memory capacity, findings consistent with extensive research on cognitive control impairments in clinical populations (Barch et al., 1997, 2000; Chatham et al., 2009). However, the methamphetamine-dependent participants (partial *η*^2^ = 0.046) exhibited more pronounced impairments than those with social media overuse (partial *η*^2^ = 0.037). This distinction is particularly meaningful given that working memory serves as the foundational resource of cognitive control, providing the necessary resources for both proactive and reactive control to operate effectively (Kane & Engle, 2002; Wiemers & Redick, 2018). The severe working memory deficits in methamphetamine users may therefore compromise their ability to flexibly engage different control strategies, potentially contributing to the greater real-world functional challenges and adverse life outcomes often observed in substance-dependent populations (Dean et al., 2013).

The key divergence between the two addiction populations emerged in the patterns of proactive and reactive control. Our componential analysis using the AX-Continuous Performance Task (AX-CPT) highlighted more profound disruptions in reactive control among Methamphetamine-dependent individuals. Specifically, these individuals struggled with ‘late-stage corrections’ (Braver, 2012), demonstrating compromised abilities to rapidly direct attention, resolve conflicts, and adapt behavior when presented with target stimuli. These deficits are consistent with known structural and functional impairments in the dorsolateral prefrontal cortex (DLPFC) and anterior cingulate cortex (ACC) following prolonged methamphetamine use (Dom et al., 2005; Moorman, 2018; Winstanley, 2007). Such impairments hinder the effective engagement of reactive control, thereby increasing susceptibility to failures in self-regulation and higher relapse rates (Sinha, 2001; Tiffany & Conklin, 2000).

In contrast, individuals with social media overuse exhibited comparatively milder deficits in overall cognitive control. Although they showed impairments in working memory capacity and trends toward reduced baseline performance and reactive control, these differences did not reach statistical significance, indicating that the extent of cognitive control and its inhibitory process impairments in this group was less pronounced. This suggests that while social media overuse may exert subtle impacts on cognitive functioning, it does not substantially disrupt cognitive control networks to the same degree as substance dependence does. Instead, it primarily affects reward-related circuitry, such as the ventral striatum and orbitofrontal cortex (Alavi et al., 2012; Grant et al., 2010; Kuss & Griffiths, 2012; Robbins & Clark, 2015; Sherman et al., 2018).

Further differentiating these two addiction profiles, our analysis of the Proactive Behavioral Index (PBI) revealed distinct reliance patterns. Methamphetamine-dependent individuals displayed a maladaptive control profile, overly relying on an already compromised reactive control system instead of attempting to bolster proactive strategies. This heavy dependence on impaired reactive control likely stems from underlying prefrontal dysfunction that hinders the effective utilization of more adaptive, anticipatory control processes (Miller & Cohen, 2001). Conversely, individuals who overuse social media did not exhibit significant differences in their cognitive control patterns relative to the control group. Although social media overusers showed overall impairments in cognitive control resources, their proactive control and pattern (as reflected by PBI), remained largely comparable to those of the control group. This indicates that unlike substance addiction—which directly impairs cognitive control mechanisms—excessive social media use has a relatively minor impact on cognitive control execution, resulting in less severe and more circumscribed deficits (Braver et al., 2009; Friedman & Robbins, 2022; Ryman et al., 2019).

These findings contribute to the ongoing debate about the similarities and differences between substance and behavioral addictions (Alavi et al., 2012; Grant et al., 2010; Kuss & Griffiths, 2012, 2017; Robbins & Clark, 2015). While both share impairments in cognitive control resources, the patterns and severity of these deficits diverge. Substance addiction, represented here by methamphetamine dependence, involves more pervasive disruptions—particularly in reactive control—potentially reflecting direct structural and functional damage to key neural substrates (Frazer et al., 2018). By contrast, the impairments associated with social media overuse appear more circumscribed, with proactive and reactive control relatively preserved but subtly weakened.

These distinctions underscore the need for tailored intervention strategies. For individuals with methamphetamine dependence, interventions may prioritize strengthening reactive control, as impairments in this domain can hinder their ability to respond adaptively to changing demands and increase susceptibility to relapse. Approaches such as cognitive-behavioral therapies and executive function training targeting flexible response adjustment and inhibitory control may be beneficial. In contrast, for individuals with social media overuse, interventions may focus on enhancing working memory and attentional control to support overall cognitive functioning, given the subtle deficits observed in these areas while their proactive and reactive control remain largely intact.

Several limitations should be acknowledged. Due to practical constraints, only male participants were included in the methamphetamine-dependent and corresponding control groups, limiting the generalizability of these findings. Moreover, the inherent differences in addiction severity between the substance and behavioral addiction samples may pose challenges for direct comparisons. Future research should aim for more balanced gender distributions and consider employing neuroimaging techniques to elucidate the specific neural pathways underlying these observed cognitive control impairments.

## 5. Conclusions

This study employed two experiments to compare cognitive control mechanisms in methamphetamine-dependent individuals and those who overuse social media. While both groups exhibited general impairments in cognitive control resources, their patterns of inhibition-based cognitive control deficits differed, with methamphetamine-dependent individuals showing a greater reliance on compromised reactive control and social media overusers maintaining relatively intact cognitive control execution. These findings support the notion that substance and behavioral addictions may stem from distinct underlying mechanisms, underscoring the need for tailored intervention strategies that address the specific cognitive control vulnerabilities of each population.

## Figures and Tables

**Table 1:** Demographic information and clinical characteristics of participants in Experiment 1.

	MDG ^a^	MCG ^a^	SMG ^a^	SCG ^a^
Sample Size	43	43	40	73
Gender	all males	all males	19 males 21 females	43 males 30 females
Age	35.30 (4.93)	24.12 (2.36)	18.93 (0.13)	19.10 (0.15)
Years of Education	11.43 (1.93)	13.24 (1.23)	13.11 (1.62)	14.01 (1.35)
Duration of Drug Use (years)	10.20 (4.52)	/	/	/
Monthly drug use (g)	9.19 (8.05)	/	/	/
Adapted IAT score	/	/	74.53 (0.80)	36.73 (0.87)
Adapted BFAS score	/	/	66.05 (1.09)	30.48 (0.73)

^a^ represents the mean (standard deviation); MDG and MCG refer to the methamphetamine-dependent group and its matched control group, while SMG and SCG represent the social media overuse group and its corresponding control group.

**Table 2:** Demographic information and clinical characteristics of participants in Experiment 2.

	MDG ^a^	MCG ^a^	SMG ^a^	SCG ^a^
Sample Size	43	38	42	55
Gender	all males	all males	11 males 31 females	38 males 17 females
Age	34.10 (6.43)	19.45 (1.57)	19.16 (1.46)	19.06 (1.11)
Years of Education	11.12 (1.63)	13.33 (1.21)	13.47 (1.32)	13.89 (1.15)
Duration of Drug Use (years)	7.59 (3.81)	/	/	/
Monthly drug use (g)	7.58 (9.09)	/	/	/
Adapted IAT score	/	/	72.59 (5.32)	37.50 (6.81)
Adapted BFAS score	/	/	65.01 (7.17)	30.69 (6.40)

^a^ represents the mean (standard deviation); MDG and MCG refer to the methamphetamine-dependent group and its matched control group, while SMG and SCG represent the social media overuse group and its corresponding control group.

**Table 3:** Performance metrics and trial distributions for AX-Continuous Performance Test (AX-CPT).

	Key Press	Practice Trials	Pass Standard	Total Trials	Proportion of Formal Experiment	Indices
AX	J	8	6	196	70.00%	Index of habituated response
AY	K	3	2	21	7.50%	Index of reactive control
BX	K	3	2	21	7.50%	Index of proactive control
BY	K	3	2	21	7.50%	Index of baseline response levels
BZ	J	3	2	21	7.50%	Index of strategy avoidance supplementation

As the AX trials, which comprise 70% of the total trials, merely serve to establish a dominant response, the data from the AX trials were excluded from the further analysis in Experiment 2. Similarly, as the BZ trials serve as a supplementary index and not a primary measure, they were also excluded from further analysis to focus on the main indices.

## Data Availability

The data presented in this study are available on request from the corresponding author. The data are not publicly available due to participant confidentiality and institutional ethical guidelines.

## Supplementary Materials

The following supporting information can be downloaded at: https://www.mdpi.com/article/10.3390/bs15081086/s1.
