# Supplementary material for: Differential Impact of Methamphetamine Dependence and Social Media Overuse on Cognitive Control: Based on the Dual Mechanisms of Control Theory
Source: Behav Sci (Basel). 2025 Aug 12;15(8):1086. doi: 10.3390/bs15081086 (PMC12383042; doi:10.3390/bs15081086)
Supplement: Supplementary file 1 [file behavsci-15-01086-s001.zip › behavsci-3728878-supplementary.pdf]

Supplementary materials

Selection process and criteria

Social media overuse group: We administered a screening questionnaire to college students to recruit social media overuse participants and corresponding control participants. This process utilized an adapted version of Young's Internet Addiction Test (IAT) comprising 20 items on a 5-point scale. Our preliminary data (including 1059 participants) showed an average score of 56.98±13.52. Following Cudo et al. (2019), we employed the quartile method to categorize IAT scores. Participants scoring at and below the first quartile (47 points, 264 individuals) were assigned to the control group. In comparison, those scoring at and above the third quartile (66 points, 288 individuals) were classified into the social media overuse group. To ensure the reliability of the participant selection for the social media overuse group, we conducted cross-validation using the adapted Bergen Facebook Addiction Scale (BFAS, Andreassen et al., 2012), modified to reflect general social media use instead of specifically Facebook. This scale's scores served as secondary screening criteria. Participants in the social media overuse group needed to score in the top third (greater than or equal to 56 points), whereas control group scores had to fall in the bottom 33%(less than or equal to 43 points). Following this screening, 40 participants were selected for the social media overuse group, and 73 for the control group. Additionally, participants in the social media overuse group must meet the criteria of using social media at least 5 to 7 days per week, with daily usage exceeding four hours.

The adapted version of Young's Internet Addiction Test (IAT)

The Internet Addiction Test (IAT), developed by Dr. Kimberly Young in 2004, is a validated instrument designed to assess Internet addiction. Comprising 20 items rated on a Likert scale

from 0 (rarely) to 5 (always), the IAT evaluates factors such as preoccupation with the Internet, time spent online, efforts to conceal usage, emotional attachment to online activities, and their impact on daily life, including work, social interactions, and psychological well-being. The adapted Internet Addiction Test (IAT, Young, 2004) has been modified to incorporate terminology specific to social media use, as documented by de Bérail et al. (2019) and Su et al. (2021). This adaptation of the IAT demonstrates robust reliability, with a Cronbach's alpha coefficient of 0.92 in the examined sample.

The adapted Bergen Facebook Addiction Scale (BFAS)

The Bergen Facebook Addiction Scale (BFAS), devised by Andreassen et al. in 2012, is a psychometric scale designed to measure Facebook addiction. It includes six items, each reflecting a core addiction criterion: salience, mood modification, tolerance, withdrawal, conflict, and relapse. Responses are scored on a 5-point Likert scale.Utilizing the adapted Bergen Facebook Addiction Scale (BFAS, Andreassen et al., 2012), which substitutes Facebook-specific terms with general social media terminology. The adapted BFAS demonstrated high reliability upon subsequent testing, achieving a Cronbach's alpha coefficient of 0.95.

Modified AX-CPT task (Cooper et al., 2017)

The AX-CPT task, a standard measure for assessing proactive and reactive cognitive control (Cooper et al., 2017; Gonthier et al., 2016), involves participants responding to a target stimulus based on situational cues. Each trial presents a preparatory cue, followed by a target stimulus after a latency period. The original task requires participants to respond to the AX

sequence as a target and to the AY, BX, and BY sequences as non-targets. The experimental design capitalizes on the predominance of the AX sequence in trials (e.g., 70%), thereby conditioning participants to anticipate responses to both cue A and target X. The AX-CPT task's sensitivity to control patterns (Braver et al., 2012) is evident when participants, under heightened proactive control, maintain situational cues in working memory, preparing for target responses to cue A and non-target responses to cue B. Conversely, a lower proactive control stance with heightened reactive control diminishes the influence of the cue, focusing instead on the target stimulus, thus improving AY trial performance and degrading BX trial performance. The AY trial's performance thus indexes reactive control levels, while the BX trial indexes proactive control proficiency (Braver et al., 2012).

In our pilot study, we employed the original version of the AX-CPT task with a cohort of university students and observed that many participants employed a strategic approach. Specifically, upon seeing the letter 'B' as the first stimulus, they preemptively prepared to respond with a "non-target" judgment without processing the second letter, leading to an immediate reaction. This strategic response rendered the 'X' in the target trials (AX/BX/BY) ineffective, thus eliminating any performance differences across these trial types.

We modified the AX-CPT task in the main experiment to mitigate this strategic interference with the experimental outcomes. Besides the "AX" letter pair serving as a target in this adapted version, another target pair "BZ" was introduced. Thus, regardless of the first letter, if the second letter was 'Z', participants were required to make a target judgment (notably, the "AZ" combination did not occur, only "BZ"). This adjustment ensured that participants could not prepare a "non-target" response upon merely seeing the letter 'B' (non-'A'), as the

determination depends on the second letter's appearance. This modification preserved the core logic of the original experimental manipulations without affecting its integrity. Finally, the adapted task consisted of 280 trials, structured across seven blocks with 40 trials per block, with AX trials constituting 70% of the total (196 trials), and the remaining trial types (AY, BY, BX, BZ) each comprising 7.5% (21 trials each).
